# Cervical Cancer in Botswana: Current State and Future Steps for Screening and Treatment Programs

**DOI:** 10.3389/fonc.2015.00239

**Published:** 2015-11-03

**Authors:** Surbhi Grover, Mmakgomo Raesima, Memory Bvochora-Nsingo, Sebathu P. Chiyapo, Dawn Balang, Neo Tapela, Onyinye Balogun, Mukendi K. A. Kayembe, Anthony H. Russell, Barati Monare, Senate Tanyala, Jailakshmi Bhat, Kealeboga Thipe, Metlha Nchunga, Susan Mayisela, Balladiah Kizito, Ari Ho-Foster, Babe Eunice Gaolebale, Ponatshego A. Gaolebale, Jason A. Efstathiou, Scott Dryden-Peterson, Nicola Zetola, Stephen M. Hahn, Erle S. Robertson, Lilie L. Lin, Chelsea Morroni, Doreen Ramogola-Masire

**Affiliations:** ^1^Department of Radiation Oncology, Perelman School of Medicine, University of Pennsylvania, Philadelphia, PA, USA; ^2^Botswana University of Pennsylvania Partnership, Gaborone, Botswana; ^3^National Cervical Cancer Prevention Programme, Ministry of Health, Gaborone, Botswana; ^4^Department of Oncology, Gaborone Private Hospital, Gaborone, Botswana; ^5^Princess Marina Hospital, Gaborone, Botswana; ^6^Division of Global Health Equity, Brigham and Women’s Hospital, Boston, MA, USA; ^7^Department of Radiation Oncology, New York University Perlmutter Cancer Center, New York, NY, USA; ^8^National Health Laboratory, Gaborone, Botswana; ^9^Department of Radiation Oncology, Massachusetts General Hospital, Boston, MA, USA; ^10^Department of Medicine, Perelman School of Medicine, University of Pennsylvania, Philadelphia, PA, USA; ^11^Division of Infectious Diseases, Brigham and Women’s Hospital, Boston, MA, USA; ^12^Division of Radiation Oncology, University of Texas MD Anderson Cancer Center, Houston, TX, USA; ^13^Department of Microbiology, Perelman School of Medicine, University of Pennsylvania, Philadelphia, PA, USA; ^14^School of Medicine, University of Botswana, Gaborone, Botswana; ^15^Institute for Women’s Health, University College London, London, UK; ^16^Institute for Global Health, University College London, London, UK; ^17^Department of Obstetrics and Gynecology, Perelman School of Medicine, University of Pennsylvania, Philadelphia, PA, USA; ^18^Department of Obstetrics and Gynecology, Faculty of Medicine, University of Botswana, Gaborone, Botswana

**Keywords:** cervical cancer, Botswana, cancer screening, cancer treatment, HIV and cancer

## Abstract

Botswana has a high burden of cervical cancer due to a limited screening program and high HIV prevalence. About 60% of the cervical cancer patients are HIV positive; most present with advanced cervical disease. Through initiatives by the Botswana Ministry of Health and various strategic partnerships, strides have been made in treatment of pre-invasive and invasive cancer. The See and Treat program for cervical cancer is expanding throughout the country. Starting in 2015, school-going girls will be vaccinated against HPV. In regards to treatment of invasive cancer, a multidisciplinary clinic has been initiated at the main oncology hospital to streamline care. However, challenges remain such as delays in treatment, lack of trained human personnel, limited follow-up care, and little patient education. Despite improvements in the care of pre-invasive and invasive cervical cancer patients, for declines in cervical cancer-related morbidity and mortality to be achieved, Botswana needs to continue to invest in decreasing the burden of disease and improving patient outcomes of patients with cervical cancer.

## Introduction

Cervical cancer is the second most common cancer in women in low- and middle-income countries (LMICs), with an estimated 530,000 new cases each year. More than 270,000 women die from cervical cancer annually, with >85% of these deaths occurring in LMICs. Botswana, a former British protectorate in Sub-Saharan Africa, is primarily an English speaking democracy of 2 million people with a stable political environment. Within Botswana, cervical cancer is the leading cause of cancer death. More than two-thirds of cases occur in HIV-infected women (1), with a national HIV prevalence of 17–24% in 2013 (2)^1^^,^^2^. Between 2003 and 2011, cervical cancer accounted for 14% of all cancers in Botswana and 26% of all the cancers in women, and the mean age at presentation was 52 years. Over 250,000 women in Botswana are in the age group 30–49 years about 25% of total female population (2) and are, thus, at high risk of developing cervical cancer^3^. Among this subset of women, the HIV prevalence rate approaches 50%. The number of women at-risk of cervical cancer will continue to grow until effective primary prevention efforts are established (3).

Several strategic partnerships and initiatives over the last decade have played a pivotal role in making cervical cancer prevention a national priority in Botswana. The main catalyst for these collaborations has been the cervical cancer prevention pilot program funded by the President’s Emergency Plan for AIDS Relief (PEPFAR). Over the last 5 years, data from this pilot program have informed national cancer prevention policy (4), and guided the Ministry of Health’s (MOH) ambitious plan to provide comprehensive, organized cervical cancer prevention services nationally^3^.

The purpose of this piece is to provide an overview of current situation of cervical cancer screening and treatment programs in Botswana, to describe successes and challenges of the cervical cancer programs and highlight partnerships that have been significant to development of cervical cancer screening and treatment programs in Botswana.

## Screening

Organized screening, public awareness, and early treatment of pre-cervical cancer lesions have led to the relatively low incidence of cervical cancer in high-income countries (5). The high incidence and mortality rate from cervical cancer in LMICs could be reduced by effective screening and treatment programs (6)^4^. Cytology testing using Pap smear has been the mainstay of screening in high-income countries (7); however, use of cytology screening in LMICs has proven difficult given limited resources for pathology review with resultant delays in diagnosis and limited treatment access leading to disease progression (8, 9). The Government of Botswana has recognized the burden of disease that cervical cancer poses, and, as a result, for the last two decades, has attempted to provide screening in the form of cytology. Unfortunately, these programs have had a limited impact on cervical cancer incidence and mortality due to challenges with follow-up, as well as pathological and treatment capacity in a setting where screening results are positive for a high proportion of women.

“See and Treat” (SAT) screening, as an alternative to cytology, is performed through visual inspection after acetic acid (VIA) application to the cervix followed by immediate treatment with cryotherapy for screen positive patients. The evidence-based SAT approach has been implemented in low-resource settings around the world (10–12). Several South African studies have found the SAT approach cost-effective, safe, and efficacious in screening for and treating cervical dysplasia (13, 14). SAT procedures have been endorsed by multiple international organizations, including The American Congress of Obstetrics and Gynecology and The Royal College of Obstetricians and Gynecologists (15).

Following the success of the PEPFAR-funded SAT pilot program initiated by the Botswana-UPenn Partnership (BUP) in 2009, the Botswana MOH endorsed VIA in 2011 as an additional screening modality, linked to same treatment visit cryotherapy. The first comprehensive national cervical cancer prevention strategic plan (2012–2016) incorporated the lessons learned from this pilot. The strategy calls for the dual use of VIA and Pap smear (use of either methods) for wider screening coverage for women aged 30–49 years (the highest at-risk group for pre-cancer). Implementation of this strategy, in part, will be made possible by additional PEPFAR funding through the pink ribbon red ribbon (PRRR) initiative of the Bush Foundation. PRRR is a public/private partnership between the United States government and private organizations for the prevention of breast and cervical cancer. With PRRR support, Botswana has set up dual VIA/cryotherapy services linked to colposcopy/loop electrical excisional procedure (LEEP) clinics in five facilities using the template developed through the SAT pilot. The LEEP clinics will also provide service for patients referred with abnormal Pap smear results. These five facilities are within the catchment area of 80% of the at-risk women. The target is to screen around 40,000 (combined Pa and VIA) each year. Current annual screening numbers are around 30,000 smears and 3500 VIA a year. Service is provided by nurses and non-specialized doctors at VIA and LEEP clinics respectfully. Quality assurance and mentoring is maintained by an expert team through monthly visits and review of cervical images from the clinics.

There is planning for future addition of HVP DNA point-of-care testing as a primary screening method once it is available and affordable. This is because the HPV DNA testing’s sensitivity is more superior to both cytology and VIA testing, and there is possibility of using self-collected swabs for screening. The HPV screen positive patients will be provided SAT in the established clinics.

As Botswana’s cervical cancer-screening program evolves, robust histopathology services will play an integral role in the successful management of women with pre-cancerous and cancerous disease of the cervix. Challenges surrounding the lack of availability of pathology services in sub-Saharan Africa, Botswana included, are numerous and impact negatively on patient care. These obstacles include inadequate infrastructure, limited personnel (pathologists and technicians), limited training opportunities, and poor funding for pathology services (16).

Fortunately, the MOH has recognized the need to substantially improve the anatomic pathology infrastructure in order to support the SAT program and the rising burden of cancers in general. Since 2009, the volume of specimens has doubled in Botswana, in part due to the successes of the SAT program, but the lab infrastructure had also eroded due to equipment problems and lack of sufficient personnel. With consultative input and in-kind donations from external partners [American Society for Clinical Pathologists (ASCP), BUP, and Botswana Oncology Global Outreach (BOTSOGO)], the MOH developed a comprehensive plan to expand capacity and decrease delays. In the past year, the National Heath Laboratory has installed an automated tissue processor and slide-stainer and tripled the number of functioning microtomes, eliminating the several week backlog resulting from a scarcity of histology technicians and insufficient equipment. Additionally, the laboratory has installed an automated high-throughput slide scanner to enable telepathology as a strategy to address the shortage of pathologists in Botswana. The University of Botswana initiated a pathology residency program in January 2013 and these residents will further expand the pool of qualified pathologists. Finally, during periods of extended backlog, the MOH has utilized the private sector to expedite timely pathology results.

## Cervical Cancer Vaccination

Cervical cancer will remain as a difficult problem unless an effective HPV vaccine program is introduced for primary prevention (17)^3^. To this end, the Botswana MOH has added nationwide HPV vaccination of 9- to 13-year-old girls, about 10% of female population, to the current cervical cancer prevention efforts. A comprehensive HPV vaccination implementation plan for the national HPV vaccine roll out in 2015, funded by government of Botswana, using vaccine doses at a negotiated price from the manufacturer, has been completed and will be operationalized through the MOH’s Expanded Program for Immunization (EPI). Two doses of quadrivalent vaccination will be given to girls only. The target group in the first year will be all girls in standards/grades 5–7 (last 3 years in primary school), and all 9–13 years old for girls not in school. In subsequent years, the vaccination will be for grade 5 girls in school and 9-year-old out-of-school girls (about 24,000 girls, about 2% of the female population, which should be a manageable number). The vaccination campaign will be conducted twice a year. The first dose was given the week of February 23rd 2015 and the second dose will be given within 6–12 months. During vaccination weeks, vaccination is carried out in all public and private schools around the country, and the local clinics cater to the out-of-school girls.

Built into this geographic expansion and intensification of cervical cancer prevention is the aim of collecting quality data that can be used to evaluate the program at the end of the strategy period (2016). Furthermore, these data will allow locally relevant evidence to inform the second comprehensive cervical cancer prevention strategy document for the period of 2017–2021.

## Cervical Cancer Treatment

Even if these prevention interventions were implemented at scale today in Botswana, they would not have impact on the large burden of cervical cancer for 10–20 years. It is, thus, vital that efforts also prioritize earlier detection and treatment of invasive cancer.

The majority of patients in Botswana present with locally advanced disease at diagnosis (1) when the primary treatment includes radiation therapy and cisplatin-based chemotherapy, rather than surgery (18). There are no gynecological oncologists in Botswana. Patients suspected of having cervical cancer are generally referred to gynecology for biopsy and pathological confirmation. A baseline evaluation is performed, including routine laboratories, chest x-ray, and renal ultrasound, if there is a concern for hydronephrosis. If the disease is locally advanced, the Botswana government sponsors patients to receive radiation therapy (external beam and brachytherapy) at Gaborone Private Hospital (GPH), the only radiation facility currently in the country.

For an estimated population of 2 million, there is a single radiation oncology facility in Botswana with one linear accelerator and three radiation oncologists, located at the GPH which, although private, sees and treats 95% of the patients diagnosed with cervical cancer from public hospitals in the entire country (19, 20). Approximately 65 patients per day are treated at GPH on a single machine (19) and about 200 cervical cancer patients are treated per year at GPH with chemoradiation. Brachytherapy, internal radiation crucial to treatment of cervical cancer, was initiated at GPH in 2012 (21). For cervical cancer, unlike other solid malignancies in Botswana, all aspects of care, including chemotherapy and radiation, are delivered at GPH. Over 95% of patients treated at GPH are referred from government hospitals. Although the government covers all aspects of their treatment at GPH, this “public guarantee” runs out on the last day of their treatment. As a result, outcomes of treatment, including effectiveness and toxicities, are currently unknown as no routine follow-up care is performed.

The MOH recognizes the importance of developing a well-coordinated multidisciplinary cervical cancer care program. However, currently, the system is fragmented with large distances and barriers to communication between diagnostic facilities, pathology laboratories, and the single comprehensive treatment center. Moreover, there are no provisions made in the current system for post-treatment follow-up of patients. As part of the University of Botswana Academic Hospital currently under construction, a multidisciplinary cancer center in the public-sector is expected to open, including pathology, and radiation, gynecological, and medical oncology services. Recently, a multidisciplinary gynecological oncology clinic has been initiated at Princess Marina Hospital (PMH), the main public-sector tertiary hospital with specialty care, to improve gynecological oncology care in Botswana. A number of external partners [UPENN, BOTSOGO, National Cancer Institute (NCI), Center for Disease Control and Prevention, and MD Anderson Cancer Center] work to support the MOH and University of Botswana to improve cervical cancer care. Important among these many initiatives is the support of a US-trained radiation oncologist (Surbhi Grover) in Botswana, expansion of research capacity through a consortia grant focused on the immunology of HIV-associated cervical cancer (U54CA190158), ongoing bidirectional exchanges and mentorship to expand local clinical capacity, and maintenance of a research cohort permitting longitudinal assessment of cervical cancer outcomes.

## Challenges and Future Directions

At present, there are limited data available regarding the stage of presentation for cervical cancer patients. A standardized and inter-linked hospital-based cancer registry representing various departments and hospitals that captures all cancer patients presenting at all stages of disease would be valuable. This registry would be crucial to understanding the burden of cancer in the country and guiding resource allocation, and would contribute toward national efforts to develop a national population-based cancer registry. Current MOH developments in this area include

- Publication and distribution of a standardized cancer (passive) reporting log book to major centers treating cancer
- Addition of cancer stage (TNM) to the metrics being collected in the registry
- Training in May 2015 to cancer clinicians and registrars, and there is advocacy to expand the number of registrars who are conducting active reporting into the registry (currently, there are two for the entire country).

Based on pilot data from BUP, we know that patients undergoing radiation therapy present mostly with stage IIB or higher disease, but we are unaware of both how many patients are presenting with early stage disease that may be treated in other gynecology facilities around the country, and also trends in disease stage at presentation. This information is crucial to understanding the impact of both screening and vaccination programs, especially as the screening programs are being expanded in an effort to downstage cervical cancers.

Patient education and community sensitization are crucial for timely diagnosis and treatment. Expansion of the screening program provides a unique opportunity for patient education regarding the goals of screening, signs of disease, and the opportunities to access care if disease is suspected. These education efforts will also help with delays in treatment and may lead to fewer cases of advanced stage at diagnosis.

Based on pilot data from BUP (1), we know that over 60% of cervical cancer patients with advanced disease are HIV positive. However, randomized studies that established the standard of care for cervical cancer did not include any patients with HIV. We need additional studies to understand the outcomes of cervical cancer patients in Botswana, and to validate the current standard of care or establish a new standard of care for this HIV-infected patient.

Based on pilot data, the median time from diagnosis to treatment is about 4–5 months (1). Currently, it is unclear why that may be, although potential hypotheses include the distance from treatment center, poor healthcare systems, easier access to traditional healers, financial opportunity costs, limited cancer awareness, and cancer stigma. Better understanding of the relevant contributors to delay will be crucial to improving cancer-related mortality. Similarly, identifying and addressing the barriers to continue follow-up is essential for early detection and intervention for disease recurrence and management of treatment side effects. This comprehensive approach will lead to both improved patient outcomes and quality of life.

Lack of access to trained human resources is another challenge. Appropriate cancer care requires adequate resources in pathology, radiology, oncology nursing, and specifically for radiation, therapy-medical physicists, radiation therapy technicians, and dosimetrists. MOH is aware of these challenges and is making efforts toward tackling these issues with various domestic and international stakeholders.

In conclusion, the national cervical cancer screening and vaccination programs have the potential to positively impact the plight of cervical cancer patients in Botswana. Several efforts are underway to improve treatment for women with cervical cancer. Moreover, the experience and data obtained from these efforts can inform ongoing as well as future domestic and international collaborations that will expand capacity to prevent, treat, and study cervical cancer. We hope that some of the lessons learned in Botswana can be used in the future to develop a sustainable and comprehensive oncology program in other countries as well.

## Conflict of Interest Statement

The authors declare that the research was conducted in the absence of any commercial or financial relationships that could be construed as a potential conflict of interest.

## Footnotes

^1^Botswana S. *Preliminary Results Botswana AIDS Impact Survey IV (BAIS IV) 2013* (2013).

^2^*HIV and AIDS Estimates [Webpage]* (2013). Available from: http://www.unaids.org/en/regionscountries/countries/botswana

^3^Five-year comprehensive preventions and control strategy (2012-2016). In: *National Cervical Cancer Prevention Program*. Republic of Botswana Ministry of Health (2012).

^4^Human papilloma and cervical cancer. In: *Fact Sheet* (2013). Available from: http://www.who.int/mediacentre/factsheets/fs380/en/
